# Sexual Differences in response to Mid- or Low-Premixed Insulin Analogue in Patients with Type 2 Diabetes

**DOI:** 10.1155/2020/8152640

**Published:** 2020-02-07

**Authors:** Bing-li Liu, Xiao-mei Liu, Yi-fei Ren, Yi-Xuan Sun, Meng-hui Luo, Lei Ye, Jian-hua Ma, Feng-fei Li

**Affiliations:** ^1^Department of Endocrinology, Nanjing First Hospital, Nanjing Medical University, Nanjing, China; ^2^National Heart Research Institute Singapore, National Heart Centre Singapore, Singapore

## Abstract

**Objective:**

To observe whether there are sexual-related differences in response to mid- or low-premixed insulin in type 2 diabetic patients.

**Methods:**

This was an analysis of CGM data of a previous study. After screening, patients with longstanding T2D receive a 7-day continuous subcutaneous insulin infusion (CSII) therapy, and then subjects were randomly assigned 1 : 1 into two groups receiving Novo Mix 30 or Humalog Mix 50 regimen for a 2-day phage, followed by a 4-day cross-over period. A 4-day continuous glucose monitoring (CGM) was performed during the cross-over period. The primary endpoint was the differences in glycemic control between male and female patients receiving mid- or low-premixed insulin therapy.

**Results:**

A total of 102 patients (52 men and 50 women) completed the study. Our data showed that male patients had significant decrease in mean glucose levels monitored by CGM after three meals during Humalog Mix 50 treatment period compared to those received Novo Mix 30 regimen (0900: 11.0 ± 2.5 vs. 12.2 ± 2.8, 1000: 9.9 ± 2.9 vs. 11.3 ± 3.1, 1200: 8.0 ± 1.9 vs. 9.1 ± 2.5, 1400: 9.2 ± 2.3 vs. 10.3 ± 2.5, and 2000: 7.3 ± 2.1 vs. 8.2 ± 2.4 mmol/L, *p* < 0.05, respectively). In addition, male patients receiving Novo Mix 30 experienced a significantly increased hypoglycemic duration compared to those of receiving Humalog Mix 50 (0 (0, 4.8) vs. 0 (0, 0), *p* < 0.05, respectively). In addition, male patients receiving Novo Mix 30 experienced a significantly increased hypoglycemic duration compared to those of receiving Humalog Mix 50 (0 (0, 4.8) vs. 0 (0, 0),

**Conclusion:**

Our data indicate that male patients with T2D receiving mid-premixed insulin analogue regimen may have a potential benefit of improvement in glycemic control compared to female patients. This trial is registered with ClinicalTrials.gov ChiCTR-IPR-15007340.

## 1. Introduction

Premixed insulin analogues are an optional choice for patients with type 2 diabetes (T2D) to maintain their blood glucose concentrations in the target range [1]. Although no guidelines recommended for initiation or intensification of premixed insulin analogues, patients who received basal insulin regimen and failed to achieving glycemic control can benefit from thrice-daily premixed insulin analogue intensified therapy for a 24-week treatment [2]. Furthermore, a phase 4 randomized trial reported that low- or mid-premixed insulin analogue as insulin initiations showed similar effect on improving glycemic control in patients with T2D. However, researchers found that more patients receive mid-premixed insulin regimen achieving target HbA1c levels than those of low-premixed insulin analogue [3], which indicated that mid-premixed insulin may have potential effect on long-run glycemic control on some patients with special characteristics.

We previously reported that older male patients with newly diagnosed T2D have an increased nocturnal hypoglycemia during continuous subcutaneous insulin infusion therapy [4]. Furthermore, we demonstrated that male patients with longstanding T2D need lower total, basal, and bolus insulin doses to maintain their blood glucose control compared to those of female patients. Importantly, male patients had a significantly increased incidence of hypoglycemia during intensive insulin therapy [5]. Our results were in accordance with others reporting that gender/sex differences play a role in glucose lowering therapy for type 2 diabetes (T2D) [6–8]. Specially, estradiol levels were positively correlated to insulin resistance in female patients with T2D [9], but not in male [9, 10]. We hence hypothesized that male and female patients may have different responses to mid- or low-premixed insulin analogues with regard to glycemic variations in patients with T2D. We used continuous glucose monitoring (CGM) to monitor glucose profile as CGM provides rigorous 24 h glucose profiles [11, 12].

Therefore, a post hoc analysis, by using CGM data in patients with T2D in whom treated with Mix 30 or Mix 50 regime, comparing sex-related differences in response to mid- or low premixed insulin analogue was performed.

## 2. Methods

**Figure 1:**
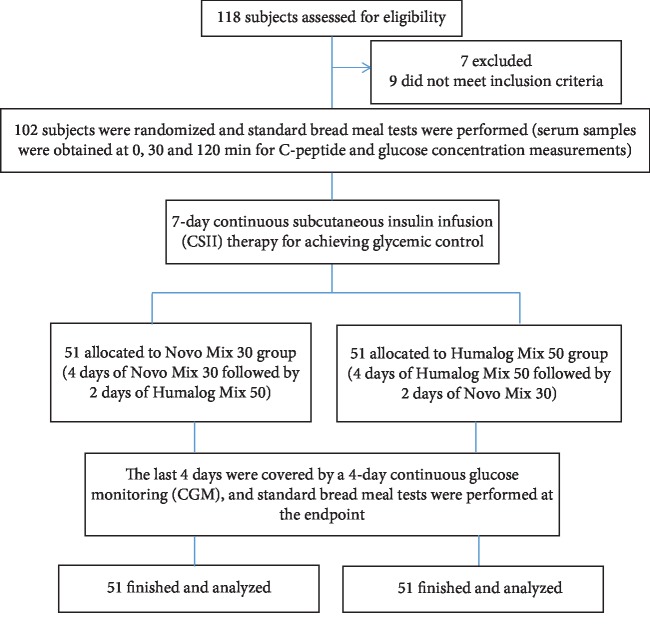
Study flow chart.

This was an analysis of CGM data of a previous study [5] (ClinicalTrials.gov, number ChiCTR-IPR-15007340). The study protocol and patient consent forms were approved by the Institutional Ethical Committee of Nanjing First Hospital, Nanjing Medical University. All procedures were in accordance with the ethical standards of Nanjing First Hospital and with the Helsinki Declaration of 1964 as revised in 2013. Informed consent was obtained from all patients recruited in the study. The study flow chart was described as Figure 1.

The study was performed at the Department of Endocrinology, Nanjing First Hospital, Nanjing Medical University, and the study period was from February 2013 to December 2014. The study includes a screening period, a 7-day run-in period, followed by a 6-day cross-over period. We enrolled patients with persistent hyperglycemia after receiving oral antihyperglycemic agents at least 3 months. The inclusion criteria also included patients with age at 18-80 years old and HbA1c levels 7.5-12%. The excluded criteria were patients positive for antiglutamic acid decarboxylase antibody or if they had maturity onset diabetes in the young (MODY) or mitochondria diabetes mellitus [13].

After screening, the demographic data were collected from all enrolled subjects by two independent specialist researchers, and a standard bread meal test (100 g) was performed by nurses at day 1 of the study [14]. Venous blood samples were obtained at 0, 30, and 120 min after meal loading, then were centrifuged, and stored at -80°C until C-peptide, HbA1c, and glucose concentrations were centrally measured.

The recruited subjects received CSII therapy from day 2 to day 9, as previously described [12, 15, 16]. Then, patients were randomly assigned 1 : 1 to 2 groups receiving (1) 4 days of Novo Mix 30 thrice daily (Novo Nordisk, Bagsværd, Denmark) followed by 2 days of Humalog Mix 50 thrice daily (Humalog® Mix 50™, Eli Lilly and Company, IN, USA); (2) 4 days of Humalog Mix 50 thrice daily followed by 2 days of Novo Mix 30 thrice daily. The premixed insulin analogues were injected three times 30 minutes before each meal, and insulin doses were calculated by the total daily insulin doses of the end of the CSII therapy, and dose titration was according to capillary glucose values obtained by self-monitoring. Subjects did not receive any oral antihyperglycemic agents during the study period. Investigators titrated insulin doses on an individual-patient basis at the titration algorithm (if the blood glucose level was less than 4.4 mmol/L, the basal insulin dose was reduced 2 units; if the blood glucose level was within 4.4 to 6.1 mmol/L, the basal insulin dose was unchanged; if the blood glucose level was within 6.2 to 7.8, 7.9 to 10.0, and >10.0 mmol/L, the basal insulin dose was increased subsequently by 2, 4, and 6 units, respectively), as described previously [5].

The 4-day retrospective CGM device at the cross-over period was Sof-sensor, CGMS-Gold, Medtronic Incorporated, Northridge, USA. During CGM period, all subjects were instructed to maintain moderate physical activity and three meals consisting of a total daily caloric intake of 25 kcal/kg/day (ratio of carbohydrate, proteins, and fats was 55%, 17%, and 28%, respectively) were served at 0700, 1100, and 1700, respectively [17]. In addition, minor meals or snacks were permitted when patients had hypoglycemic symptoms during the 4-day CGM period.

The trial was cross-over design feature, so the 24 hr washout period should be fully taken into account [18]. We therefore collected the day 2 and day 4 CGM data to analyze the whole 48 hr glycemic variations when patients receive mid- or low-premixed insulin analogues. In detail, the mean postprandial glucose concentrations of each meal, the 24 hr mean glucose concentrations (MG), the standard deviation of 24 hr MG (SDMG), the 24 hr mean amplitude of glycemic excursions (MAGE) [4, 19], the incremental area under curve (AUC) of hyperglycemia (>10.0 mmol/L), and the incremental area over curve (AOC) of hypoglycemia (<3.9 mmol/L) were calculated, respectively.

The primary outcome was the differences in glycemic control between patients receiving mid- or low-premixed insulin therapy. The secondary endpoints were the differences in the glycemic profiles between the two groups.

### 2.1. Statistical Analysis

Normal distribution data were presented as the means ± SD, and nonnormal distribution data were presented as median with quartile range. Statistical analysis was performed using SPSS software (version 17.0; SPSS, Inc., Chicago, IL). The Shapiro-Wilk test was used to assess the distribution of data and the Wilcoxon test was employed to compare nonnormal distribution data. A chi-squared test was performed comparing the ratio differences between two groups. The mixed ANOVA model (2 × 2) test was used to compare differences between groups. A two-way ANOVA was used for repeated measurements for the group comparisons, followed by the Bonferroni-Dunn post hoc test. *p* values were two-tailed with a significance level of 5%.

## 3. Results

### 3.1. Baseline Characteristics

A total of 118 subjects with T2D were assessed for eligibility between February 2013 and December 2014 in Department of Endocrinology, Nanjing First Hospital, Nanjing, China. Sixteen patients were excluded because of 7 patients with glucose levels above 22.2 mmol/L and 9 patients who did not meet inclusion criteria (Figure 1). The CGM data of 102 (52 men and 50 women) patients collected at the endpoint were analyzed. The demographic characteristics of the enrolled subjects were described in detail (Table 1). During the 4-day cross-over study period, patients needed 0.7 ± 0.3 IU/kg∗day premixed insulin analogues to maintain glycemic control.

### 3.2. 24 hr Glycemic Variation Profiles

**Figure 2:**
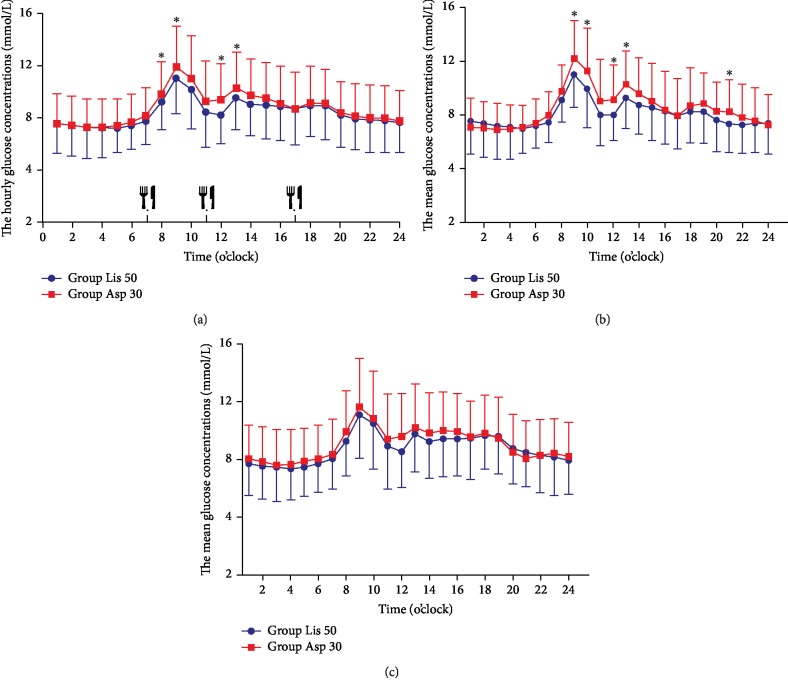
The average blood glucose concentrations per hour in all patients. ^∗^*p* < 0.05.

A total of 102 patients completed the study yielding 832 (range 332-860) glucose readings per patient. Our CGM data showed that subjects receiving Novo Mix 30 had the similar glycemic variations, such as MAGE, SD, CV%, the incremental AUC of hyperglycemia, and the incremental AOC of hypoglycemia compared to those receiving Humalog Mix 50 therapy (*p* > 0.05) (Table 2). We also analyzed CGM data per hour to compare fasting and postprandial glucose levels between the two groups. Our data showed that patients treated with the two types of premixed insulin analogues had the same fasting glucose concentrations at the cross-over period. Interestingly, we observed that patients receiving Humalog Mix 50 had significantly decreased mean glucose concentrations after breakfast and lunch compared to those with Novo Mix 30 therapy (0800: 9.8 ± 2.4 vs. 9.2 ± 2.1, 0900: 11.9 ± 3.1 vs. 11.0 ± 2.8, 1200: 8.2 ± 2.3 vs. 9.4 ± 2.8, and 1300: 9.5 ± 2.5 vs. 10.3 ± 2.7 mmol/L, *p* < 0.05, respectively) (Figure 2(a)). In addition, our data showed that patients receiving Novo Mix 30 had significantly increased in duration of hypoglycemia and TIR compared with those whom receiving Humalog Mix 50 group (Table 2). We further observed that male patients receiving Novo Mix 30 showed significant increase in duration of hypoglycemia and TIR than those of whom receiving Humalog Mix 50 group, while female patients had the similar response to Novo Mix 30 or Humalog Mix 50 in terms of duration of hypoglycemia (Table 3).

### 3.3. Sexual Response Different to Mid- or Low-Premixed Insulin Analogues in Glycemic Control

A stratified analysis was performed to determine whether male or female patient response is different to mid- or low-premixed insulin analogues with regard to glycemic variations and hourly glucose levels. Our data showed that male and female patients exhibited the similar MAGE, SD, CV%, the incremental AUC of hyperglycemia, and the incremental AOC of hypoglycemia between the Novo Mix 30 and Humalog Mix 50 group at the endpoint (Table 3). However, our data clearly showed that male patients had significant decrease in mean glucose levels monitored by CGM after three meals during Humalog Mix 50 treatment period compared to those received Novo Mix 30 regimen (0900: 11.0 ± 2.5 vs. 12.2 ± 2.8, 1000: 9.9 ± 2.9 vs. 11.3 ± 3.1, 1200: 8.0 ± 1.9 vs. 9.1 ± 2.5, 1400: 9.2 ± 2.3 vs. 10.3 ± 2.5, and 2000: 7.3 ± 2.1 vs. 8.2 ± 2.4 mmol/L, *p* < 0.05, respectively) (Figure 2(b)). However, the same findings were not observed in female patients (Figure 2(c)).

### 3.4. Sexual Differences in Hypoglycemia

In addition, there were no differences in the incremental AOC of hypoglycemia (defined as glucose less than 3.9 mmol/L delivered by CGM) in patients receiving either Novo Mix 30 or Humalog Mix 50 (Table 2). However, we observed a significant increase in hypoglycemic duration in patients with Humalog Mix 50 therapy compared to those who received Novo Mix 30 therapy (0 (0, 2) vs. 0 (0, 0), *p* < 0.05). A stratified analysis indicated that female patients had similar hypoglycemic duration between the both groups (0 (0, 0) vs. 0 (0, 0), *p* > 0.05). However, male patients receiving low-premixed insulin analogues experienced a significantly increased hypoglycemic duration compared to those of receiving mid-premixed insulin analogues (0 (0, 4.8) vs. 0 (0, 0), *p* < 0.05). There were no severe hypoglycemia events reported during the whole study period.

## 4. Discussion

Premixed insulin analogues showed effectiveness in improvement of postprandial glucose levels than long-acting insulin regimens for containing component controlling postprandial blood glucose concentrations [20, 21]. Premixed insulin analogue regimens are recommended to China patients as initial insulin therapy [2] partially may be due to the factor that isolated postprandial hyperglycemia is more prominent in Chinese patients when compared to white patients [22, 23]. In this study, we expected to see a significant decrease in postprandial glucose levels in patients receiving Humalog Mix 50 regimen compared to Novo mix 30 twice daily therapy.

Our data showed that longstanding T2D patients may have a potential benefit in improvement of postprandial glucose levels receiving mid-premixed insulin analogue compared to those of low-premixed insulin analogue therapy. Our data indicate that male patients may be the population who benefit better compared to female patients.

Studies highlighted that sex plays an important role in glucose-lowering regimen [6–8]. Our data further indicated that sex may play a role in response to premixed insulin analogue regimen, with regard to postprandial glucose levels. In agreement with our previous study reporting that men may have an increase risk in nocturnal hypoglycemia [4] and an increased incidence of hypoglycemia during intensive insulin therapy [5], our data showed that male patients receiving mid-premixed insulin analogue achieved a potential improvement in postprandial glucose concentration compared to those of female patients, while female patients did not have any difference in postprandial glucose levels with mid- or low-premixed insulin analogue. Therefore, a compressive understanding for gender-specific glucose-lowering strategy is desirable.

In this study, the recruited male subjects have the following characteristics: age was 58.6 years, body mass index (BMI) was 24.2 kg/m^2^, HbA1c value was 10.2%, and course of disease was 6.2 years, which were all similar to those of the enrolled female patients. It is unknown why male patients had a better response to mid-premixed insulin analogue compared to low-premixed insulin analogue regimen. However, given our cross-over study design feature, we can exclude many confounding factors which might weaken our findings.

We observed that men needed significantly lower Novo Mix 30 or Humalog Mix 50 doses compared to female patients at the endpoint. However, our data showed that male patients receiving Novo Mix 30 had higher incidence of hypoglycemia compared to those of receiving Humalog Mix 50 regimen. In this study, the recruited subjects with age around 60 yrs. old, together with our previously published study, report that only patients above 60 yrs. old had the sex difference in incidence of hypoglycemia [4]. However, this situation might not suitable for younger T2D male patients.

Other limitations should also be addressed. Firstly, the study design was not considered insulin titration scope and optimization of individual therapy. Secondly, the adiposity profiles were not collected from the recruited subjects, which may contribute to the sex-related differences in response to different premixed insulin analogues. Thirdly, we had no data backing the mechanism for male patients might benefit more receiving mid-premixed insulin analogue compared to female.

In conclusion, our data indicate that male patients with longstanding T2D receiving mid-premixed insulin analogue may be potential benefit candidate for controlling better postprandial glucose levels compared to female patients.

## Figures and Tables

**Table 1:** Baseline of the recruited subjects.

Items	All	Group by sex		
	Male/female (52/50)	Male (52)	Female (50)	*p* value
Age (yrs.)	59.4 ± 11.8	58.6 ± 11.7	60.3 ± 11.9	0.47
BMI (kg/m^2^)	24.7 ± 3.8	24.2 ± 3.12	25.2 ± 4.4	0.20
Waistline (cm)	87.7 ± 8.5	87.0 ± 7.2	88.4 ± 9.8	0.46
Hipline (cm)	93.8 ± 8.3	93.5 ± 7.8	94.1 ± 8.9	0.76
Waist-to-hip ratio (%)	0.9 ± 0.1	0.9 ± 0.1	0.9 ± 0.1	0.49
TDD1 (U/kg)	0.7 ± 0.3	0.6 ± 0.2	0.8 ± 0.3	0.001^∗∗^
Bolus (U/kg)	0.3 ± 0.1	0.3 ± 0.1	0.4 ± 0.1	0.01^∗^
Basal (U/kg)	0.3 ± 0.2	0.3 ± 0.1	0.4 ± 0.2	0.001^∗∗^
TDD2 (U/kg)	0.6 ± 0.3	0.5 ± 0.2	0.7 ± 0.3	0.001^∗∗^
BI (U/kg)	0.3 ± 0.1	0.2 ± 0.1	0.3 ± 0.1	0.01^∗^
LI (U/kg)	0.2 ± 0.1	0.15 ± 0.07	0.2 ± 0.1	0.01^∗^
DI (U/kg)	0.2 ± 0.1	0.2 ± 0.1	0.3 ± 0.1	0.001^∗∗^
Duration (yrs.)	6.6 ± 6.2	6.2 ± 6.3	6.9 ± 6.2	0.57
HbA1c (%)	10.0 ± 2.2	10.2 ± 2.3	9.8 ± 2.1	0.41
C-peptide 0 (pmol/L)	1.5 ± 1.1	1.5 ± 1.0	1.5 ± 1.2	0.75
C-peptide 120 (pmol/L)	3.2 ± 2.5	2.9 ± 2.3	3.6 ± 2.6	0.16

^∗^ *p* < 0.05, male vs. female; ^∗∗^*p* < 0.01, male vs. female. BMI: body mass index; TDD: total daily dose; BI: breakfast insulin dose; LI: lunch insulin dose; DI: dinner insulin dose; C-peptide 0: C-peptide level after glucose loading at 0 min; C-peptide 120: C-peptide level after glucose loading at 120 min.

**Table 2:** Glycemic variations in patients receiving low- and mid-mixed insulin analogues.

Items	All subjects		
	Asp30	Lis50	*p* value
Duration > 10.0	27 (11, 44)	21(7.75, 37)	0.06
Duration < 3.9	0 (0, 2)	0 (0, 0)	0.03^∗^
Duration 3.9-10	67.3 ± 22.4	74.6 ± 20.3	0.01^∗^
AUC > 10.0	0.5 (0.1, 1)	0.4 (0.1, 0.8)	0.08
AOC < 3.9	0 (0, 0)	0 (0, 0)	0.34
AUC 3.9-10	20.7 ± 5.3	19.6 ± 4.9	0.13
24 hr MG	8.8 ± 1.7	8.4 ± 1.5	0.08
MAGE	5.6 ± 2.2	5.4 ± 2.1	0.56
CV%	25.9 ± 8.3	25.8 ± 8.4	0.96
SDMG	2.2 ± 0.7	2.2 ± 0.7	0.37

^∗^ *p* < 0.05, asp30 vs. lis50. Duration > 10.0: time spent in glucose levels above 10.0 mmol/L (%); duration < 3.9: time spent in glucose levels less than 3.9 mmol/L (%); AUC > 10.0: the incremental area under curve of glucose > 10.0 mmol/L (mmol/L∗day); AOC < 3.9: the incremental area over curve of plasma glucose < 3.9 mmol/L (mmol/L∗day); AUC 3.9-10: the incremental area under the curve of glucose levels between 3.9 and 10 mmol/L; 24 hr MG: 24 hr mean glucose (mmol/L); MAGE: mean amplitude of glycemic excursions (mmol/L); CV%: the coefficient of variation; SDMG: standard deviation (mmol/L).

**Table 3:** Glycemic variations in male and female patients receiving low- and mid-mixed insulin analogues.

Items	Male (52)			Female (50)		
	Asp 30	Lis 50	*p*	Asp 30	Lis 50	*p*
Duration > 10.0	25 (10.3, 38.8)	19 (6, 31.3)	0.14	34.5 (15, 50)	22 (10.8, 46.3)	0.28
Duration < 3.9	0 (0, 4.8)	0 (0, 0)	0.01^∗^	0 (0, 0)	0 (0, 0)	0.86
Duration 3.9-10	69.7 ± 21.1	79.1 ± 15.6	0.01^∗^	64.7 ± 23.7	70 ± 23.5	0.27
AUC > 10.0	0.5 (0.1, 0.8)	0.3 (0.1, 0.5)	0.13	0.7 (0.2, 1.3)	0.5 (0.1, 1.3)	0.35
AUC < 3.9	0 (0, 0)	0 (0, 0)	0.44	0 (0, 0)	0 (0, 0)	0.56
AUC 3.9-10	19.8 ± 5.0	18.5 ± 4.2	0.16	21.6 ± 5.6	20.7 ± 5.3	0.42
24 hr MG	8.5 ± 1.5	8.0 ± 1.2	0.1	9.0 ± 1.8	8.7 ± 1.7	0.35
MAGE	5.8 ± 2.2	5.6 ± 2.2	0.71	5.3 ± 2.2	5.1 ± 2.0	0.65
CV%	27.2 ± 9.0	27.1 ± 8.9	0.97	24.5 ± 7.2	24.5 ± 7.7	0.98
SDMG	2.3 ± 0.8	2.2 ± 0.7	0.45	2.2 ± 0.7	2.1 ± 0.7	0.62

^∗^ *p* < 0.05, male asp30 vs. male lis50. Duration > 10.0: time spent in glucose levels above 10.0 mmol/L (%); duration < 3.9: time spent in glucose levels less than 3.9 mmol/L (%); AUC > 10.0: the incremental area under curve of glucose > 10.0 mmol/L (mmol/L∗day); AOC < 3.9: the incremental area over curve of plasma glucose < 3.9 mmol/L (mmol/L∗day); AUC 3.9-10: the incremental area under the curve of glucose levels between 3.9 and 10 mmol/L; 24 hr MG: 24 hr mean glucose (mmol/L); MAGE: mean amplitude of glycemic excursions (mmol/L); CV%: the coefficient of variation; SDMG: standard deviation (mmol/L).

## Data Availability

All the data used to support the findings of this study are available from the corresponding author (Prof. Jian-hua Ma, email address: majianhua196503@126.com) upon request.
